# On the need to adjust for multiplicity in confirmatory clinical trials with master protocols

**DOI:** 10.1093/annonc/mdz038

**Published:** 2019-01-31

**Authors:** N Stallard, S Todd, D Parashar, P K Kimani, L A Renfro

**Affiliations:** 1 Statistics and Epidemiology, Warwick Medical School, University of Warwick, Coventry; 2 Department of Mathematics and Statistics, University of Reading, Reading; 3 The Alan Turing Institute, London; 4 Warwick Cancer Research Centre, University of Warwick, Coventry, UK; 5 Division of Biostatistics, University of Southern California, Los Angeles, USA

Recent advances in tumor biology and targeted therapies have led to clinical trials assessing treatment efficacy in multiple patient populations or subgroups using a *master protocol* [1]. These can lead to efficiency gains by testing several statistical hypotheses in the same clinical trial.

Although much of the development of novel design approaches has been in exploratory phase II trials that may not include a control group, there is growing interest in such methods in confirmatory randomized controlled trials. These might be phase III trials with subgroup analyses or phase II/III trials combining exploratory and confirmatory elements. In these settings, multiple hypothesis tests can lead to statistical error rate inflation. A recent Food and Drug Administration draft guidance [1] notes the risk of ‘potential overinterpretation of findings’, but offers no clear suggestions as to when statistical correction for multiplicity should be implemented.

Before considering multiplicity adjustment, we first describe some recently used innovative designs. Our aim is not to give a comprehensive review, but to illustrate key relevant concepts. Based on the principle that correction is necessary when there are multiple opportunities to make a (regulatory) claim of efficacy for a particular treatment in any population or subgroup [2, 3], we propose when corrections are needed if the innovative designs are used in confirmatory trials.

Interest in stratified medicine has led to *subgroup selection*, or *treatment interaction*, trials [4] comparing one or more experimental treatments with a control treatment in a population comprising two or more pre-defined subgroups, for example based on biomarker levels. Such trials assess treatment efficacy in the whole population and the subgroups.

Examples of these trials include the Gefitinib versus docetaxel in previously treated non-small-cell lung cancer (INTEREST) trial [5] that assessed non-inferiority in the overall population and superiority in high epidermal growth factor receptor (EGFR)-gene-copy patients, and the Biomarker-integrated Approaches of Targeted Therapy for Lung Cancer Elimination 1 (BATTLE 1) trial [6], a phase II trial comparing four non-small-cell lung cancer treatments in five biomarker-defined subgroups to determine the best treatment for each subgroup.

Subgroup selection trials may also include interim analyses to decide whether recruitment should continue from the whole population or be restricted to one or more subgroups where the experimental treatment appears most effective, as in an *adaptive enrichment design* [7–9].

When experimental treatment options depend on one or more biomarkers within a single cancer type, several treatments may be evaluated in parallel in an *umbrella trial* [1]. If each experimental treatment is evaluated in a single subgroup, the parallel sub-studies are essentially independent trials, each considering one treatment in its own distinct patient group, conducted together for administrative or operational reasons such as saving time, reducing costs or facilitating recruitment.

The sub-studies, which are often randomized, could be two-arm comparisons, each comparing one experimental treatment to a control, or multi-arm comparisons of several drugs within each sub-study. An example of the former is FOCUS4: a molecularly stratified trial programme in colorectal cancer [10] which included randomized sub-studies of a different experimental treatment in each of five biomarker-defined subgroups, with early stopping for futility. The Biomarker-Driven Protocol for Accelerating Development of Therapies for Squamous Cell Lung Cancer (Lung-Map) trial [11] has a similar design, with different treatments assessed in parallel in marker-specific subgroups.

The development of therapies targeting a particular mutation that may be present in multiple tumor types has led to the *basket trial* design.

Some authors [1, 12] use this term for a trial in which patients with a common mutation are recruited from populations with different tumor types. The effect of the experimental treatment can then be assessed both in the whole recruited group and in the individual tumor types. Often such trials are small and non-randomized, with all patients receiving the experimental treatment.

An example of such a trial is the imatinib study B2225 [13]. This open-label phase II trial investigated the effect of imatinib in patients expressing imatinib-sensitive tyrosine kinases in 40 different life-threatening malignancies, including solid tumors and hematologic malignancies.

The term *basket trial* has also been used to describe a more complex trial in which recruited patients have different tumor types and different mutations [14]. Patients are then assigned to experimental treatments according to their mutation. Acknowledging that this type of design extends the simpler definition to include more than one mutation type, such a trial has also been called a *matrix trial*, although this term has also been used more generally, including both basket and umbrella trials as special cases.

An example of a matrix trial is the National lung matrix trial [15] in which seven drugs are evaluated in 18 targeted molecular cohorts, with drugs evaluated in one to five cohorts.

Multiplicity issues may arise when multiple hypothesis tests are conducted, particularly in confirmatory trials. Conventionally, adjustments are made when multiple confirmatory hypothesis tests are conducted in a trial [16], but not when they are conducted in separate trials. This position has arisen mainly in simpler settings than those now being developed, and the use of innovative trial designs has led to reconsideration of multiplicity issues, with little current consensus [2, 3, 17].

In the confirmatory setting, a basic principle is that correction for multiplicity is needed if testing of multiple hypotheses, whether through testing in multiple subgroups or at interim analyses, leads to multiple opportunities to inform a single claim of effectiveness for a drug [2].

**Figure 1.**
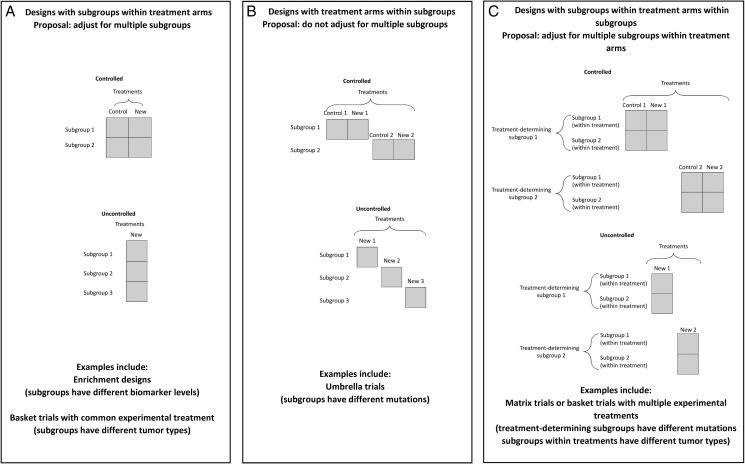
Designs for clinical trials with master protocols: (A) with subgroups within treatment arms; (B) with treatment arms within subgroups; (C) with subgroups within treatment arms within subgroups.

In considering multiplicity issues in trials testing hypotheses based on different subgroups, as in the examples above, it is helpful to differentiate between trials on the basis of the role of the subgroups. We will consider three cases illustrated in Figure 1 and discussed in detail in the next three paragraphs.

We use the term *subgroups within treatment arms* to describe a setting in which the population is divided into subgroups, but in which the subgroup classification does not determine the experimental treatment options. In a non-randomized setting, all patients receive the same experimental treatment. In a randomized setting, all patients are randomized between the same two or more treatments, usually including a control (Figure 1A). The trial then assesses the effect of the same experimental treatment(s) in a number of subgroups. In this setting, since patients in different subgroups can receive the same experimental treatment(s), testing might also be conducted with subgroups combined as well as in individual subgroups.

This situation may be contrasted with one in which we have *treatment arms within subgroups*, when the population is divided into subgroups with distinct experimental treatment options available to each. In a non-randomized trial, the treatment is determined by the subgroup classification, and in a randomized trial, the treatments available for randomization depend on the subgroup so that randomization can only be undertaken for a patient once their subgroup is known (Figure 1B). The trial then assesses the effects of a number of experimental treatments, each in its corresponding subgroup.

An extension of the two settings above occurs when a trial includes subgroups of both types; in this case, subgroups are defined in two ways, with one of these restricting experimental treatment options, giving *subgroups within treatment arms within subgroups* (Figure 1C). An example is a situation in which patients are classified by two biomarkers, biomarker 1 and biomarker 2, with the experimental treatment depending on biomarker 1 but not on biomarker 2. Patients with a positive biomarker 1 status might then be allocated to treatment A, or randomized between A and a control treatment C, and biomarker 1 negative patients be allocated to treatment B or randomized between B and C, in each case irrespective of biomarker 2 status. The trial then assesses treatment A in two subgroups of biomarker 1-positive patients who are either positive or negative for biomarker 2 and treatment B in two subgroups of biomarker 1-negative patients who are either positive or negative for biomarker 2.

Our aim is to correct for multiplicity when multiple hypothesis testing increases the chance of erroneously declaring a given ineffective treatment to be effective in all or some part of the population, but not for multiple hypothesis testing for different treatments when the use of each is restricted to a single subgroup. Using the definitions introduced above, we therefore propose that multiplicity corrections should be used in the setting with *subgroups within treatment arms*, or for the *subgroups within treatment arms within subgroups*, including the case of testing in combinations of subgroups, but not with *treatment arms within subgroups*.

Our rationale is that with *subgroups within treatment arms*, the same treatment is evaluated in a number of subgroups, giving multiple opportunities to claim this treatment is effective. This contrasts with the *treatment arms within subgroups* setting, where each experimental treatment is considered for only one subgroup so that there are not multiple opportunities for an effectiveness claim for any particular treatment.

The following paragraphs illustrate the application of this proposed multiple testing strategy in the designs introduced previously.

In subgroup selection, treatment interaction or adaptive enrichment designs the aim is to assess the experimental treatment(s) in the whole population and/or in one or more subgroups. As treatment options are not determined by subgroup classification, we have *subgroups within treatment arms*. In a confirmatory trial setting, our proposal is therefore to adjust for multiplicity arising from hypothesis testing in multiple subgroups in such a trial, for example using a Holm–Bonferroni correction [18], or a method allowing for overlapping subgroups [19].

In umbrella trials, we have *treatment arms within subgroups*. Our proposal would therefore be to not adjust for multiplicity arising from the testing of multiple hypotheses in different subgroups. This proposal is consistent with the view that the sub-studies, though run under a single protocol, are essentially independent trials, as each subgroup receives a different experimental treatment so that the efficacy of each treatment is assessed only once.

In the simpler type of basket trial [12] one experimental treatment is evaluated in patients with different tumor types. We thus have *subgroups within treatment arms*. In a confirmatory trial, we would therefore propose adjustment for multiplicity arising from hypothesis testing in the different tumor types to protect the overall chance of erroneously claiming effectiveness of the experimental treatment.

In matrix trials or the more complex type of basket trial with distinct treatments tested in different mutations [14], we have *subgroups* (e.g. tumor types) *within treatment arms within subgroups* (e.g. mutations). In confirmatory trials, we would therefore propose adjustment for the multiple hypothesis tests of the effectiveness of the same treatment in different tumor types, but not for testing in different mutations since mutation determines treatment.

Although we have focused on specific trial settings, the principles are generalizable. For example, the same design might be used if subgroups are specified not by a molecular biomarker but by another measurable patient characteristic that determines treatment. Similarly, the same design might be used in a trial not just with multiple tumor types, but also with subgroups defined by some other factor in which we are trying to ascertain subgroups for which a treatment is effective, requiring multiplicity to be handled in the same way. Consideration of the same concepts could also enable application of the novel trial designs in other therapeutic areas.

We have focused on multiplicity arising from multiple subgroups. Trials, including platform or multi-arm multi-stage trials, may also include multiple treatments, possibly in addition to multiple subgroups. Multiplicity issues in such settings are discussed elsewhere [3, 17]. The need for multiplicity correction depends on the relatedness of the hypotheses being tested. If different doses of the same drug are tested, multiplicity corrections will be more appropriate than when multiple arms are unrelated treatments, possibly with different modes of action, whether or not they are compared with a common control group. Error rates other than familywise error rates, including false discovery rates and family multiple error rates, could also be considered in such settings [3].

Multiplicity arising from testing a single treatment in a number of different indications could also be considered, as could methods for testing a treatment strategy using data from multiple subgroups each receiving a different experimental treatment [20]. 
